# A new innovative method to measure the cost of war: future with fewer conflicts via harm reduction approaches

**DOI:** 10.1186/s12962-024-00517-4

**Published:** 2024-01-30

**Authors:** Ehsan Jozaghi

**Affiliations:** Faculty of Dentistry, University of British Columbia, 2199 Wesbrook Mall, Vancouver, BC V6T 1Z3 Canada

**Keywords:** Military, War, Peace, WWI, WWII, Harm reduction, Global warming, Free trade, Diplomacy, The UN, The NATO, The World Trade Organization, The World Bank

## Abstract

**Background:**

The destruction of World War I (WWI) and World War II (WWII) changed the world forever. In this analysis, the economic costs of WWI and WWII are considered via a harm reduction approach to highlight the cost of war via the mortality of military personnel. The harm reduction philosophy and homeostasis of a biological cell are utilized as a pragmatic approach and analogy to give a greater context to the findings, despite the omission of civilian casualties and military disabilities.

**Methods:**

Tangible (e.g., loss of wages, productivity, and contributions) and intangible (e.g., quality of life) costs are estimated based on the value of each military personnel derived from secondary data and a mathematical model. This is the first study to estimate the cost of war based on soldier’s mortality during the first and second World War.

**Results:**

Based on the tangible value, the WWI and WWII cost for the military personnel was US$43.204 billion ($13 billion ≤ *α* ≤ $97 billion) and US$540.112 billion ($44 billion ≤ *α* ≤ $1 trillion). When the intangible cost is considered, it is estimated that the WWI cost was beyond US$124 trillion ($43 trillion ≤ *β* ≤ $160 trillion), and the WWII cost was above US$328 trillion ($115 trillion ≤ *β* ≤ $424 trillion). The sensitivity analyses conducted for WWI and WWII demonstrate different ranges based on tangible and intangible values.

**Conclusions:**

In the current climate of increasing hostilities, inequalities, global warming, and an ever-changing world, economic prosperities are directly linked to peace, stability, and security. Therefore, any future decisions for military conflicts need to increasingly consider harm reduction approaches by considering the cost of life and potential disabilities for each nations’ soldiers, sailors, and pilots.

## Introduction

The global community has come a long way from the destruction of World War I (WWI) and World War II (WWII). The global community is advancing with new scientific discoveries in medicine, astrophysics, artificial intelligence (AI), and engineering. Some have described the current century as the “scientific golden age”, as the frequency of new discoveries is rapidly changing nature and the limits of our understanding [1]. In comparison to previous centuries, the internet, social media, free-trade, technological breakthroughs, and increasingly affordable modes of transportation have allowed us to be connected socially, culturally, geographically, and economically like never before. Advances in science and medicine have allowed us to save lives and recover quickly from a global pandemic that would have wiped out populations to the scale of plague events in past centuries [2].

Development of free trade within nations has capitalized on many nations’ economic capabilities, providing economic efficiencies, creating jobs, generating wealth and prosperities, linking continents, all while preventing war via economic integration. Furthermore, investments via trade have been able to lift millions of people from poverty, giving access to clean water, affordable foods, education, medicine, the internet, and stable sources of income. Increasingly, the concepts of money lending by the World Bank and non-government agencies play a significant role in linking economies, reducing poverty, instability and ensuring peace within nations.

Lamentably, the recent hostilities in Eastern Ukraine, the Middle East, north and central Africa, the South China Sea, and the Korean peninsula are destabilizing the peace, economic prosperity, and security of many nations. Global warming is also adding significant strains to geopolitical norms. Poverty, in addition to a lack of access to clean water and basic life necessities, could pose significant challenges in the future. In this climate of increasing hostilities and geopolitical instabilities, how can the global community ensure prosperity, peace, and security? What can nations do to ensure that the golden age of economic prosperity and scientific discoveries continue their extraordinary pace into the twenty-second century?

Looking at past conflicts through new perspectives would allow nations to consider new approaches for preventing war and ensuring peace in the twenty-first century. Consider the current conflicts in Syria, for example; in many ways, cities such as Aleppo remind us of the sheer destruction of the historic World Wars in Europe and Asia. While most politicians, government policy makers, diplomats, and researchers have focused solely on the human tragedies of war, such as the mass destruction of cities, numerous civilian causalities, and mass refugee migrations, it is essential to highlight the cost of war through a harm reduction perspective.

## Harm reduction

Harm reduction has been an evidence-based approach to health care where more pragmatic avenues are considered to reduce harm to patients. The harm-reduction approach traces its history to the HIV outbreak of the 1980s where a community driven, practical, and pragmatic approach based on the principles of low-barrier access to public health was conceived [3]. The harm reduction philosophy is based on the Ottawa Charter for Health Promotion aimed at establishing and improving access and availability of care so that an immediate risk of harm is mitigated [3].

These risk reduction efforts have played major roles in the public health policies of many nations for centuries; for example, the malaria reduction strategies via the draining of swamps in ancient Rome, harm reduction strategies for HIV prevention amongst marginalized populations, and current air pollution reduction strategies [4–6]. More recent developments in the realm of harm reduction includes supervised consumption facilities that prevent drug overdose deaths while simultaneously linking clients to health care professionals such as nurses, social workers, and doctors [3]. The above noted risk/harm reduction practices have demonstrated effective public health approaches for preventing premature death, disability, and disease [4–6].

Therefore, a harm reduction approach can be effectively employed in future diplomatic negotiations, conflicts, and war where the security of both civilian populations and military personnel are considered in the cost–benefit evaluations. The harm reduction approach in the context of military, economic, and diplomatic policy making could promote not only a cost-effective remedy but also a more rational and humane choice for the future.

This harm reduction approach would entail placing a monetary value on the lives and potential disability of each military personnel to reduce the most destructive harm to civilians and military members. In the same way that overdose prevention sites deter imminent death while allowing the client to continue the path of self-recovery at their own pace, the harm reduction approach also allows nations to reduce imminent death and destruction via less lethal means, such as trade embargos, military alliances, sanctions, diplomatic isolation, no-fly-zones, blockades, economic-scientific de-coupling and limited strikes.

Without the harm reduction philosophy and the lack of knowledge about the true cost of military conflicts during the actual outbreaks of wars, major military conflicts such as another world war cannot be averted. However, when policymakers use economic evaluations via a harm reduction approach, greater emphasis is placed on prevention, containments, and diplomatic/trade re-engagements to reduce the possibility of miscalculations and most significant casualties. In other words, harm reduction philosophy is based on rational choice theory, where policymakers, diplomats and military leaders rely on rational considerations (e.g., weighing consequences and potential benefits), even in circumstances such as war, to minimize the potential casualties and destructions.

Therefore, if we consider war in the same context of harm reduction and rational choice, war is a rational act driven by a desire. In effect, the desire in war (e.g., territory gains or staying in power for a dictator by diverting the public opinion from real issues facing the nation) is the force which urges a politician to initiate a conflict: the pain of the punishment via harm reduction approaches (e.g., trade-embargos, sanctions, diplomatic isolation, blockades, no-fly-zones, military alliances, economic-de-coupling and limited strikes) is the force utilized for restraints. If the first of these forces is greater, the act of war may initiate; if the second, the war will not. Similarly, no nation undertakes war without the hope of gain, although such gain may be seen as irrational in the current century. Therefore, harm reduction allows many nations to prevent direct conflict and another major world war via less lethal interventions. In effect, harm-reduction approaches, although with different titles/names, have been able to keep the peace since the Second World War, with only a handful of international wars in the past century.

## Costing evaluation and rationale

Cost–benefit analysis and economic evaluations are a well-developed methodology that has been utilized by government and policy makers for decades [7]. Many government agencies now require costing studies as part of their decision-making process for major projects that require extensive investment/spending. Costing studies have helped governments to make critical decisions that could affect the environment, health, and safety of their citizens. Unlike environment, health, and safety evaluations, costing studies to start or end military conflicts are complex and in large part impossible to formulate because there has not been enough data to do so, or such decisions are part of national security. This is especially important when the value of life is considered for the healthiest, most courageous, and the brightest young men and women who gave the ultimate sacrifice during the First and the Second World Wars as soldiers, sailors, and pilots.

Considering harm reduction philosophy via costing evaluations, the policymakers could ask: Is war/military conflict the solution in every circumstance, and at what cost? Policymakers can answer such pragmatic questions via scientific methods or AI. Using AI may allow diplomats and military leaders to consider less costly lethal means in significant issues or military conflicts. In other words, understanding war via a harm-reduction evaluation lens and developing ways to thwart it requires concepts and models that rely on human rationality (such as harm reduction-rational choices), which can be used to account for the non-uniform, non-randomness and irrational patterns that characterize the outbreak of war.

Similarly, simplified methods that accounted for the reliable number of deaths needed to be employed to account for war's non-uniform and non-random nature. While there have been estimates of civilian causalities in the past, there are more reliable and accurate numbers of military casualties because of the reliability of military records. The model and methods employed in this study used parameters available on the World Wide Web and a reliable model used in previous harm reduction studies to facilitate valid estimation. Therefore, the current analysis attempts to evaluate WWI and WWII via an economic perspective in the context of a harm reduction-rational approach to give military leaders and diplomats realistic cost scenarios when faced with decisions to prevent, manage, and reduce the potential of military conflicts in the future.

## Methods

### Model

The current analysis relied on a model that could reflect the loss of life due to military conflicts, only the tangible value of life to society is considered rather than evaluating the suffering and lost quality of life for the military families. The values consider the tangible value by calculating the hypothetical value of the remaining lifetime wages of an average person from the community during 1914–1918 and 1939–1945. The military personnel who died during WWI and WWII were estimated to be 24.25 (average age) [8] and 26 (average age) years old [9, 10].

To place an economic value on deaths, the study conducts two analyses: 1- Only the tangible cost is considered; 2- Tangible and intangible values are calculated. To consider the tangible costs, the most direct measure of tangible costs is the average potential value that a person may contribute to the economy if they had been alive. The discount rate of 3% is used to account for future earning loss [11]. Therefore, this converted 45 years of future wages for WWI and 40 years for WWII to present value using a standard discount rate when considering that the average retirement age was 70 during 1918 and 66 after WWII [12, 13].

The average income during the WWI period varies from high-income countries, such as the UK at US$10,000 per family household, compared to low-income countries, such as India at around US$1,400 [14]. Therefore, the average value of $4,444 is utilized for the model below [14]. During WWII, the US man’s average individual income was US$39,600 [15] while an Indian worker’s annual salary was US$1,733.50 [16]. Therefore, $21,000 is the average value.

### Variables and parameters

The value of a single prevented loss of death (*V*) is calculated based on the model below:

$$V\, = \,\sum\nolimits_{i\, = \,1}^{N} {\frac{W}{{\left( {1\, + \,r} \right)\,i}}}$$

where *N* represents the remaining years of income, *W* represents the median wage for both military personnel during WWI or WWII, and *r* represents the discount rate. The sources and values for the mathematical formula above are given in Table 1 below.

**Table 1:** Variables and the sources utilized for the model and tables

Variable	Value	Note	Source
Estimated value of tangible death averted (*V*) for WWI	$194,155	Calculated via the model	
Estimated value of tangible death averted (V) for WWII	$917,476	Calculated via the model	
Average years until retirement for WWI (*N*)	45	Assuming retirement age of 70	McQuilton [8]
Average years until retirement for WWII (*N*)	40	Assuming retirement age of 66	Kennett [9]; U.S. Wings [10]
The average wage for military personnel during WWI (*W*)	$4,444	Estimated from the lower and upper limits	Broadberry & Harrison [14]
The average wage for military personnel during WWII (*W*)	$21,000	Estimated from the lower and upper limits	Department of Labor [15]; Palekar [16]
Represents the discount rate (*r*)	3%	The value of future wages lost discounted at 3%	Laufer [11]
WWI military deaths	Total: 9,721,937	Table 1 data	Mougel [39]; Britannica [38]
WWII military deaths	Total: 25,719,600	Table 2 data	The National WWII Museum [37]

The above model has been utilized in numerous economic evaluation studies [17–19]. However, if the value of life is estimated based on the contingent valuation method (CVM) or “willingness to pay”, which includes tangible (e.g., loss of wages, productivity, and contributions) and intangible (e.g., quality of life) costs[7]. CVM is a survey-based approach where consumers are asked to place a monetary value on goods that are not normally traded as commodities [20]. These commodities might include crime victimizations, clean air, clean water, and even quality of life. It is frequently used in benefit–cost evaluations in a variety of disciplines [7]. For intangible estimates derived from the CVM method, the contingent evaluation methods are used for evaluations, the lower range value of US$4.5 million has been estimated in the previous CVM literature [7]. The upper range value of US$16.5 million has been estimated in the literature [7]. Therefore, the amount of US$12.78 million is used for the baseline calculation [7].

### Sensitivity analysis

A sensitivity analysis was conducted to evaluate the reliability of the model for both the tangible and intangible costs. The sensitivity analysis for tangible estimates relates to simulating different scenarios for the annual income. Convincingly, the finding for both the baseline and sensitivity analysis for WWI and WWII costs establishes the reliability of estimates. The high-income annual salary of US$10,000 (e.g., the UK) per family household during WWI is used for upper limit of sensitivity analysis, while a salary of US$1,400 (e.g., India) is used for a lower limit of sensitivity analysis for WWI [14]. During WWII, the US$39,600 [15] individual income is used for the upper limit of sensitivity analysis, whereas US$ $1,734 is used for the lower limit [16]. The sensitivity analysis for intangible estimates included US$4.5 million as the lower bound estimates and US$16.5 million for the upper limit estimates based on the literature on the CVM [7].

### Validity and reliability

Validity and reliability are essential concepts in any research. Reliability is an assessment of reproducibility, while validity is an assessment of the results' intended measurements. In the current research, the validity and reliability of data were linked to the mathematical model and the reliability of parameters. For validity, Eddy [21] concluded that "there is no simple and universally applicable procedure for validating a model," and each model must be evaluated in the context of the study. However, Eddy [21] recommended transparency regarding the model's structure, data sources, the study research question, and sensitivity analysis. At the same time, Buxton et al. [22] and Sheldon [23] recommended the reliability of the model based on five criteria: (1) simplicity of the model; (2) transparent of the results; (3) quality of the data utilized; (4) sensitivity analysis usage to account for any uncertainties; and (5) comparing the model against other costing studies. As discussed previously, the current study has relied on a model which is not only simplified but has been used to estimate prevented overdose deaths in other harm reduction studies [19]. The parameters used in the current model are based on the World Wide Web available to the public with appropriate citation of each parameter used for transparency. The use of military deaths, when compared to civilian deaths, is a more accurate estimate because of the reliability of archives that go back more than 100 years. The research question aligns with the model utilized, and the sensitivity analysis accurately portrays the upper and lower potential parameters.

## Results

In addition to highlighting the number of causalities for each country, the total number of soldiers who died during WWI and WWII as shown in Tables 2 and 3 are 9,721,937 and 25,719,600 respectively.

**Table 2:** The total number of soldiers, sailors, and pilots killed during WWI and the cost evaluation in addition to the sensitivity results for the tangible and intangible cost for WWI

Name of the countries from 1914 to 1918	WWI	Estimates based on tangible values	Estimates based on intangible values
Austria-Hungary	1,100,000	$4.888 billion ($1.54 billion; $11 billion)*	$14.058 trillion ($4.95 trillion; $18.15 trillion)
Australia	61,928	$275.208 million ($86.699 million; $619.280 million)	$791.439 billion ($278.676 billion; $1.022 trillion)
Bangladesh, India, & Pakistan	74,187	$329.687million ($103.862 million; $742million)	$948 billion ($334 billion; $1.224 trillion)
Belgium	58,637	$261 million ($82 million; $586 million)	$749.4 billion ($263.9 billion; $967.5 billion)
Bulgaria	87,500	$389 million($123 million; $875million)	$1.118 trillion ($393.750 billion; $1.444 trillion)
Canada & Newfoundland	66,148	$293.962 million ($92.607 million; $661.480 million)	$845.371 billion ($297.666 billion; $1.091 trillion)
France	1,397,800	$6.212 trillion ($1.956 billion; $13.978 billion)	$17.864 trillion ($6.290 trillion; $23.064 trillion)
Germany	2,050,897	$9.114 billion ($2.871 billion; $20.509 billion)	$26.210 trillion ($9.229 trillion; $33.840 trillion)
Greece	26,000	$115.544 million ($36.400 million; $260 million)	$332.280 billion ($117 billion; $429 billion)
Italy	651,000	$2.893 billion ($911.400 million; $6.510 billion)	$8.320 trillion ($2.930 trillion; $10.742 trillion)
Japan	415	$1.844 million ($581thousand; $4.150 million)	$5.304 billion ($1.868 billion; $6.848 billion)
Kingdom of Serbia	275,000	$1.222 billion ($385 million; $2.750 billion)	$3.514 trillion ($1.238 trillion; $4.538 trillion)
Ottoman Empire	771,844	$3.430 billion ($1.1 billion; $7.72 billion)	$9.864 trillion ($3.473 trillion; $12.735 trillion)
Montenegro	3,000	$13.332 million ($4 million; $30 million)	$38.340 billion ($13.500 billion; $49.500 billion)
New Zealand	18,050	$80.214 million ($25.270 million; $181 million)	$230.679 billion ($81.225 billion; $297.825 billion)
Portugal	7,222	$32 million ($10 million; $72.22million)	$92.297 billion ($32.5 billion; $119.163 billion)
Romania	250,000	$1 billion ($350 million; $2.5billion)	$3.2 trillion ($1.125 trillion; $4.124 trillion)
Russian Empire	1,811,000	$8 billion ($2.5 billion; $18 billion)	$23.144 trillion ($8.149 trillion; $29.881 trillion)
South Africa	9,463	$42 million ($13.248 million; $94.630 million)	$120.937billion ($42.583 billion; $156.14 billion)
United Kingdom & Ireland	885,138	$3.933 billion ($1.239 billion; $8.851 billion)	$11.312 trillion ($3.983 trillion; $14.605 trillion)
United States of America	116,708	$518 million ($163 million; $1.167 billion)	$1.491 trillion ($525 billion; $1.925 trillion)
Total	9,721,937	$43.204 billion ($13.610 billion; $97.219 billion)	$124.246 trillion ($43.750 trillion; $160.412 trillion)

^*^The values in parentheses represent sensitivity analysis results

**Table 3:** The total number of soldiers, sailors, and pilots killed during WWII and the cost evaluation in addition to the sensitivity results for the tangible and intangible cost for WWII

Name of the countries from 1939 to 1945	WWII military deaths	Estimates based on tangible values	Estimates based on intangible values
Albania	30,000	$630 million * ($52 million; $1.2 billion)	$383.400 billion ($135 billion; $495 billion)
Australia	39,800	$836 million ($69.013 million; $1.576 billion)	$508.644 billion ($179.1 billion; $656.700 billion)
Austria	261,000	$5.481 billion($453 million; $10 billion)	$3.335 trillion ($1.174 trillion; $4.307 trillion)
Belgium	12,100	$254.1 million ($21 million; $479.2 million)	$154.638 billion ($54.450 billion; $200 billion)
Brazil	1,000	$21 million ($1.7 million; $39.6 million)	$12.8 billion ($4.5 billion; $16.5billion)
Bulgaria	22,000	$462 million ($38.2 million; $871.2 million)	$281.2 billion ($99 billion; $363 billion)
Canada	45,400	$953.4 million ($78.724 million; $1.798 billion)	$580.212 billion($204.3 billion; $749.1billion)
China	4,000,000	$84.000 billion ($6.936 billion; $158.4billion)	$51.120 trillion($18. trillion; $66 trillion)
Czechoslovakia	25,000	$525 million ($43.4 million; $990 million)	$319.5 billion ($113 billion $413 billion)
Denmark	2,100	$44 million ($3.641 million; $83.2 million)	$26.838 billion ($9.45 billion; $34.65billion)
Ethiopia	5,000	$105 million ($8.67 million; $198 million)	$64 billion($23 billion; $83 billion)
Finland	95,000	$1.995 billion ($164.730 million; 3.762 billion)	$1.214 trillion($427.500 billion; $1.56 trillion8)
France	217,600	$4.57 billion ($377.32 million8; $8.617 billion)	$2.781 trillion, ($979.2 billion; $3.59 trillion)
Germany	5,533,000	$116.193 billion ($9.594 billion; $219.12 billion)	$70.712 trillion ($24.899 trillion; $91.295 trillion)
Greece	35,000	$735 million ($60.7 million; $1.386 billion)	$447.3 billion ($157.5 billion; $577.500 billion)
Hungary	300,000	$6.300 billion($520.200 million; $11.880 billion)	$3.834 trillion ($1.350 trillion; $4.950 trillion)
India	87,000	$1.827 billion ($151 million,; $3.445 billion)	$1.112 trillion ($391.5 billion; $1.436 trillion)
Italy	301,400	$6.329 billion ($522.628 million; $11.935 billion)	$3.852 trillion ($1.356 trillion; $4.973 trillion)
Japan	2,120,000	$44.520 billion ($3.676 billion; $83.952 billion)	$27.094 trillion ($9.540 trillion; $34.980 trillion)
Netherlands	17,000	$357million ($29.478 million; $673.2 million)	$217.26 billion ($76.5 billion; $280.5billion)
New Zealand	11,900	$249.9million ($20.635 million; $471.24million)	$152.082 billion($53.55 billion; $196.35billion)
Norway	3,000	$63 million ($5.202 million; $118.8 million)	$38.34 billion ($13.5 billion; $49.5 billion)
Philippines	57,000	$1.197 billion ($98.838 million; $2.257 billion)	$728.46 billion($256.5 billion; $940.5 billion)
Poland	240,000	$5.04 billion($416.16 million; $9.504 billion)	$3.067 trillion($1.08 trillion; $3.96 trillion)
Romania	300,000	$6.3 billion($520.2million; $11.88 billion)	$3.834 trillion($1.35 trillion; $4.95trillion)
South Africa	11,900	$249.9 million ($20.635 million; $471.24 million)	$152.1 billion ($53.55 billion; $196.35 billion)
Soviet Union	10,700,000	$224.7 billion ($18.554 billion; $423.72billion)	$136.746 trillion($48.15 trillion; $176.55 trillion)
United Kingdom	383,600	$8.056 billion ($665.162 million; $15.191 billion)	$4.902 trillion($1.726 trillion; $6.329 trillion)
United States	416,800	$8.753 billion ($722.731 million; $16.505 billion)	$5.327 trillion($1.876 trillion; $6.877 trillion)
Yugoslavia	446,000	$9.366 billion($773.364 million; $17.662 billion)	$5.7 trillion ($2.007 trillion; $7.359 trillion)
Total	25,719,600	$540.112 billion ($44.598 billion; $1.019 trillion)	$328.697 trillion($115.738 trillion; $424.373 trillion)

^*^The values in parentheses represent sensitivity analysis results

Thus, the value of life saved for prevented deaths is estimated to be US$43.204 billion and US$540.112 billion. For a total value of US$583.316 billion for the tangible values. As shown in the tables, each nation’s cost is evaluated.

Moreover, it is predicted that WWI and WWII have cost the global community US$124 trillion and US$328 trillion respectively in the premature and preventable death of soldiers, sailors, and pilots when intangible values are considered. The total cost of the WWI and WWII is US$452 trillion when intangible values are estimated.

*α* is the sensitivity value range based on the tangible estimation of WWI ($13 billion ≤ *α* ≤ $97 billion) and WWII ($44 billion ≤ *α* ≤ $1 trillion). *β* is the sensitivity analysis for WWI ($43 trillion ≤ *β* ≤ $160 trillion) and WWII ($115 trillion ≤ *β* ≤ $424 trillion) estimating the intangible values.

## Discussion

As results demonstrated, the true cost of war, specifically WWI and WWII, are significantly higher than previously estimated when the military personnel’s lives and safety are taken into consideration. For example, the combined cost of WWI and WWII for military personnel was US$ 583.316 billion. When considering the intangible cost, it is estimated that the combined cost of WWI and WWII was beyond US$ 452 trillion. It is important to note that $452 trillion or $583.316 billion may not reflect the actual cost due to the omission of civilian casualties, disabilities, destruction of cities, and mass refugee displacement. In addition, the values of the current study are considered underestimated when the percentage of GDP is considered. For example, in 1914, at the onset of WWI, the US GDP was $36.8 billion ($262.8 billion between 1914 and 1918), while Britain and France GDPs were $12.4 ($82.3 billion between 1914 and 1918), and $8.8 (51.2 billion between 1914 and 1918) respectively [24]. The US GDP during 1914 was 0.14% of today's GDP, while Britain and France's were 0.39% and 0.32%, respectively [23]. Therefore, the tangible cost of WWI, for instance, consumed 10.9% of the total combined GDP of the US, the UK and France from 1914 to 1918, which would translate to a much higher value of 6.42 trillion dollars in 2022 GDP for the noted three countries [25]. This aligns with the previous estimation; for instance, the cost of the War of 1812 was relatively minor in today's dollar (about $1.6 billion) [26]. However, when considering the size of the US economy in 1812 was less than 1/1,400th of it is now, the noted war consumed more than 2% of US GDP, which equals more than $300 billion in today's value [26].

World War III has not happened, and most politicians know that repeating world wars is not a good idea without knowing the total cost. In effect, many have long held the view that the ongoing wars in Europe and the events of mass refugee displacement belong to the past and are simply 'unthinkable [27, 28]. However, the recent international postures, such as China's continued reiterating that Taiwan would "surely be reunified" and the US reclaiming the Pacific airfield for the first time since the Second World War, which launched the world's only atomic bombings, are indications of rapidly changing geopolitical landscapes [29, 30]. Also, the recent hostilities in the Middle East, Eastern Ukraine, and the Korean peninsula and the threat of global warming are indications that many challenges remain ahead. In effect, the utilization of rational harm reduction approaches (e.g., sanctions, trade embargos, diplomatic isolation, blockades, no-fly-zones, military alliances, economic/scientific-de-coupling and limited strikes), although with different titles, have been able to avert many major international wars since the WWII as a force utilized for restraints in situations of non-uniform, non-randomness and irrational patterns that characterize the outbreak of war.

While there have been numerous works devoted to the subject of how to avoid "war" (e.g., Conflict Resolution, Security Studies, and International Relations), this is the first research that has framed and conceptualized the harm-reduction-rational choice perspective while estimating the potential cost of WWI and WWII for the first time. While in the past some have questioned old military alliances, recent events and increasing globalization have highlighted the need for more multilateral cooperations, greater economic integration via free trade, and enhanced security/military alliances [31]. Potential military operations need to increasingly account for security and safety of military personnel and civilian population by politicians, diplomats, and military leaders due to the significant economic burden of war highlighted in the current study.

In effect, while the evolution of borrowing money and economic efficiency has inadvertently contributed to the formation of democracy, the functioning of the economy is directly linked to the performance of democracy/political stability and vice versa [32]. Like a living cell, where the disruption of the cell’s efficiency in producing and conserving ATP can affect cell function and survival, the disruption to the economies—that are based on trades, taxes, goods/services, lending/borrowing, antitrust laws/fair competition, protection of the intellectual property—via insecuritiesweakening of the rule of law, external threats, and most importantly war could have devastating effects in the functioning of a democracy and political stability [33].

A living cell at micro level is changed by various external and internal factors (e.g., PH levels, concentration of solutes and invading viruses), both economy (e.g., interest rates, unemployment rates, birth/death rates, immigration, and commodities prices) and democracy (e.g., lack of voting, weakening of rule of law, corruption, judicial independence, and war) can also be affected by various modulators. In the same way that a living cell is fragile and can be affected by internal (e.g., cancer via cell mutation) and external (e.g., bacteria) threats, both the economy and democracy are fragile, and their smooth functioning is linked to proactive interference of governments (e.g., the National Banks’s interest rates adjustments, government tax increases/decreases and military/security spending) and people’s participation (e.g., voting during elections, freedom of speech/press, and freedom association/assembly).

Finally, considering the homeostasis of a cell at a macro level (as the whole body/system), greater medical interventions are needed to tilt the hemostasis toward the normal range via medications (e.g., insulin, cholesterol-lowering drugs, and exercise) and surgery (e.g., heart surgery/chemotherapy) when there is imbalance of glucose (e.g., diabetes), lipids (e.g., heart disease), and lifestyle changes (e.g., cigarette smoking). Sometimes, such medical interventions are too late to save the body/system from collapsing. Similarly, when the balance of the economy is affected at the macro level via significant cost of war and associated political instability, and security issues, greater and more costly economic (e.g., food rationing during WWII in some countries) and security interventions (e.g., the state of emergency laws after the September 11th terrorist attacks) are needed to bring the country/system back to its stable/balance levels and prevent the potential collapse of democracy.

While in the body there are numerous indicators to determine the body’s potential collapse/palliative care (e.g., blood tests, edema of the tissue, skin color, and imaging), in a country, a true litmus tests to determine the point of no return may not be definitive. Therefore, to preserve the smooth functioning and the symbiotic relationships between the economy, democracy, and security, greater harm-reduction strategies are needed via a proactive approach to preserve the global peace while simultaneously focusing on balancing the incremental cost as a force utilized for restraints on issues of security, free-trade, global economic prosperities, and global military alliances.

As the global community moves into the third decade of the twenty-first century, politicians, diplomats, and military leaders should consider the past mistakes and miscalculations that have cost the global community trillions of dollars and countless human lives in international conflicts.. New strategies for peace and international security need to consider the cost of life and potential disabilities via a harm reduction perspective. The cost of life and security of soldiers, sailors, pilots and civilians in international conflicts via proactive harm/risk reduction strategies have kept the outbreak of major international wars to a minimum since the Second World War. While there is skepticism about the post-Cold-War structures, and many see the League of Nations/ the UN as a failed attempt to keep peace and order, the absence of such structures could be disastrous for public opinion/confidence and international law in an increasingly interconnected world. Therefore, some of the noted post-Cold-War governmental and non-governmental agencies (such as the U.N., the NATO, the World Trade Organization, the EU, the African Union, the Organization of American States, the Association of Southeast Asian Nations, the Commonwealth of Nations, Organisation Internationale de la Francophonie, the Arab League, the Pacific Islands Forum, the Gulf Cooperation Council, the Arctic Council, the World Bank, the International Monetary Fund, the Red Cross, the Red Crescent, and Médecins Sans Frontières) not only allow the voice of dissents in the international community but also allow the potential pursuit of the common good in an increasingly unequal world.

A proactive approach would also require greater economic integration, trade, and diplomatic engagement, military cooperations, deterrence, and intelligence. However, sometimes the proactive harm reduction approach would also equate to military operations, preemptive strikes, and more lethal force to prevent greater casualties, greater human tragedies, and economic insecurities while preserving the link/balance between the economy and democracy. It is essential to highlight that World War II may have inadvertently provided an opportunity to revive democracy in many nations that reverted to dictatorship after WWI, including Germany, Japan, Austria, and Italy. A future with fewer conflicts is attainable via harm reduction approaches when the true overall cost of conflict is considered through more collaborative investments and greater engagement/deterrence through free-trade, education, dialogue, human rights, diversity, access to health care, environmental protection, poverty reduction, and internet accessibility.

## Limitations

One of the most significant shortcomings of this paper is that it does not account for civilian causalities and disabilities. If this study had accounted for civilian deaths, disabilities, medical costs, mass refugee migrations and destruction of cities, the economic estimates would have been significantly greater than what has been recorded in the current research. However, reliable military deaths were selected due to the scarcity of reliable data and accurate estimates linked to civilian causalities. Another shortcoming of this paper is not accounting for the disabilities of military personnel due to injury sustained during WWI and WWII. For example, over 19 million military personnel were wounded in WWI at a time when shell-shock syndrome, later to be known as post-traumatic stress disorder (PTSD), had not been recognized as a legitimate disability [34, 35]. In fact, many military personnel were court martialed, and some were executed for desertion and cowardice linked to their PTSD. PTSD was not added to the American Psychological Association’s treatment manual until 1980 [36].

Moreover, many disabilities were physical in nature; in fact, 25 million military personnel were wounded in battle during WWII [37]. Also, the CVM estimates should be more conservative as they are based on ex ante estimates which are willingness to pay to stay alive, and more comprehensive values that are not normally included in value of life estimation (e.g., quality of life). Consequently, it is acceptable to concede that the CVM overestimates when compared to the tangible value estimation.

It is also important to note that military expenditures of WWI and WWII were omitted in the current study. For example, the Congressional Research Service [26] reported that military expenditures of the US government alone during WWI and WWII were US$334 billion and US$4.1 trillion in 2011 values [26]. Subsequently, the estimates reported in the current study significantly underestimate the cost of the noted wars by omitting the military expenditure.

## Conclusion

In the era of our increasingly interconnected world, where many nations are looking ahead for more prosperity and growth by working towards improving their economies and their citizens’ well-being through better jobs, health care, and transportation while improving the environment, it is surprising that some nations are still interested in expanding territories in the twenty-first century when innovations, science, technology, and entrepreneurship generate more wealth and prosperity than gaining a territory. For instance, Apple is currently valued at over $3 trillion, and only a handful of nations (e.g., the US, China, Japan, Germany, the UK and India) have a GDP larger than Apple’s combined value in the market [40]. In effect, the recent military conflicts have shown that many challenges remain, and politicians invading other countries for territorial gains have other sinister motives (e.g., staying in power by diverting public opinion from issues facing their nations). While harm reduction has been applied in the context of health care policy for decades, preventing mortalities, morbidity and other harms, its recognition and conceptualization in the context of military, trade and diplomatic policy making has also facilitated a humane, pragmatic, and rational choice since the WWII by preventing and reducing major international conflicts. A more engaged, connected, economically interlinked, educated, prosperous, environmentally sustainable, and tolerant global society is not only in the best interest of peace,security, and economy, but it is in the best interest of scientific advancement and the survival of human beings into the next century.

## Data Availability

Not applicable.

## Abbreviations

AI: Artificial intelligence
CVM: The contingent valuation method
PTSD: Post-traumatic stress disorder

## References

[1] Hockfield S (2018). Our science, our society. Science.

[2] Tognotti E (2013). Lessons from the history of quarantine, from plague to influenza A. Emerg Infect Dis.

[3] Jozaghi E (2022). The opioid epidemic: task-shifting in health care and the case for access to harm reduction for people who use drugs. Int J Health Serv.

[4] Gupta GR, Parkhurst JO, Ogden JA, Aggleton P, Mahal A (2008). Structural approaches to HIV prevention. Lancet.

[5] McNeill W (2010). Plagues and peoples.

[6] Organization for Economic Cooperation and Development (OECD) (2001). OECD environmental outlook.

[7] Cohen MA, Rust RT, Steen S, Tidd ST (2004). Willingness-to-pay for crime control programs. Criminology.

[8] McQuilton J. Enlistment for the First World War in rural Australia: the case of north-eastern Victoria, 1914–1918. J Aust War Meml, (33). 2000

[9] Kennett LB. GI: The American Soldier in World War II. University of Oklahoma Press. 1997

[10] U.S. Wings. Vietnam War Facts, Stats and Myths. https://www.uswings.com/about-us-wings/vietnam-war-facts/. Accessed 30 June 2023.

[11] Laufer FN (2001). Cost-effectiveness of syringe exchanges as an HIV prevention strategy. J Acquir Immune Defic Syndr.

[12] Government of Canada. The Canadian Government Annuities Act of 1908. 1908. https://www.historymuseum.ca/cmc/exhibitions/hist/pensions/1915-1927_e.pdf. Accessed 30 June 2023.

[13] Gendell M. Trends in retirement age by sex, 1950–2005. Monthly Labor Review. 1992.

[14] Broadberry S, Harrison M (2005). The economics of World War I.

[15] Department of Labor. Handbook of facts on Women’s workers. Bulletin of Women’s Bureau, 225. 1948.

[16] Palekar SA. Real Wages in India 1939–50. Economic Weekly, 151–160. 1957.

[17] Irwin A, Jozaghi E, Weir BW, Allen ST, Lindsay A, Sherman SG (2017). Mitigating the heroin crisis in Baltimore, MD, USA: a cost-benefit analysis of a hypothetical supervised injection facility. Harm Reduct J.

[18] Hood JE, Behrends CN, Irwin A, Schackman BR, Chan D, Hartfield K, Duchin J (2019). The projected costs and benefits of a supervised injection facility in Seattle, WA, USA. Int J Drug Policy.

[19] Irwin A, Jozaghi E, Bluthenthal RN, Kral AH (2017). A cost-benefit analysis of a potential supervised injection facility in San Francisco, California, USA. J Drug Issues.

[20] Carson RT, Hanemann WM (2005). Contingent valuation. Handb Environ Econ.

[21] Eddy D, Mosteller F (1985). Technology assessment: The role of mathematical modelling. Assessing medical technologies.

[22] Buxton MJ, Drummond MF, Van Hout BA, Prince RL, Sheldon TA, Szucs T, Vray M (1997). Modelling in economic evaluation: an unavoidable fact of life. Health Econ.

[23] Sheldon TA (1996). Problems of using modelling in the economic evaluation of health care. Health Econ.

[24] Larsen D (2021). GDP of the United States, Britain, and France, 1914–1918.

[25] Bureau of Economic Analysis. Gross domestic product, fourth quarter and year 2022. 2023. https://www.bea.gov/news/2023/gross-domestic-product-fourth-quarter-and-year-2022-third-estimate-gdp-industry-and. Accessed 1 Jan 2024.

[26] Daggett S. *Costs of Major U.S. Wars*. Congressional Research Service. 2010. https://sgp.fas.org/crs/natsec/RS22926.pdf. Accessed 1 Jan 2024.

[27] Forchtner B, Kølvraa C (2012). Narrating a ‘new Europe’: from ‘bitter past’to self-righteousness?. Discourse Soc.

[28] UNHCR. Worldwide displacement hits all-time high as war and persecution increase. 2015. https://www.unhcr.org/news/stories/worldwide-displacement-hits-all-time-high-war-and-persecution-increase. Accessed 30 June 2023.

[29] Lendon B. US Air Force to reclaim Pacific airfield that launched atomic bombings as it looks to counter China. *CNN*. 2023. https://www.cnn.com/2023/12/22/asia/us-air-force-pacific-tinian-island-airfield-intl-hnk-ml/index.html. Accessed 1 Jan 2024.

[30] Tan Y. Taiwan and China will 'surely be reunified' says Xi in New Year's Eve address. BBC news. 2023. https://www.bbc.com/news/world-asia-china-67855477. Accessed 1 Jan 2024.

[31] Gordon P, Daalder I. Trump’s biggest gift to putin qualifying and conditioning the notion of NATO’s defense guarantee is a major step on the path to abandoning it. The Atlantic. 2019. https://www.theatlantic.com/international/archive/2018/07/american-commitments-nato-trump/565601/.

[32] Baklouti N, Boujelbene Y (2018). Democracy, political stability, and economic growth: evidence from MENA countries. Int J Inform Bus Manag.

[33] Dawson JF (2021). BIOC 2580: introduction to biochemistry.

[34] DK findout. Treating the wounded. 2023. https://www.dkfindout.com/us/history/world-war-i/treating-wounded/. Accessed 30 June 2023.

[35] Hampson S. Shell shock: How much has changed in 100 years? The Globe & Mail. 2014. https://www.theglobeandmail.com/news/national/shell-shock-how-much-has-changed-in-100-years/article21498602/. Accessed 30 June 2023.

[36] US Department of Veterans Affairs. PTSD: National Center for PTSD. 2023. https://www.ptsd.va.gov/understand/what/history_ptsd.asp. Accessed 30 June 2023.

[37] The National WWII Museum. Research starters: Worldwide deaths in World War II. 2023. https://www.nationalww2museum.org/students-teachers/student-resources/research-starters/research-starters-worldwide-deaths-world-war. Accessed 30 June 2023.

[38] Britannica. Killed, wounded, and missing. 2023. https://www.britannica.com/event/World-War-I/Killed-wounded-and-missing. Accessed 30 June 2023.

[39] Mougel N. World War I casualties. 2011. https://www.census.gov/history/pdf/reperes112018.pdf. Accessed 30 June 2023.

[40] Mascarenhas R. Apple’s m-cap bigger than most countries’ GDP. The Economic Times. 2023. https://economictimes.indiatimes.com/markets/stocks/news/apples-m-cap-bigger-than-most-countries-gdp/articleshow/101557439.cms. Accessed 1 Jan 2024.

